# Diagnosis and Treatment of Dentritic Cystic Tumors of the Jaws

**Published:** 1885-10

**Authors:** John S. Smith

**Affiliations:** Lancaster, Pa.


ARTICLE IV.

### DIAGNOSIS AND TREATMENT OF DFNTRITIC CYSTIC TUMORS OF THE JAWS.

BY JOHN S. SMITH, D. D. S., LANCASTER, PA.

*Diagnosis.*—Cystic tumors may be confounded with other affections which occasion swellings about the jaws, as enchrondromata, sarcomata, and myxomata, abscesses, and the collections of fluids in the antrum. Dental alveolar abscess may be distinguished by its acute course, and when in a chronic condition by the discharge of its contents through the fistula, either upon the gum, or within the oral cavity. The tumor formed by an abscess is never so sharply definite as is the case with cysts; with dropsy of the antral cavity the distention of the facial wall of the jaw is more uniform than it is with cysts.

In some cases of cystic tumors, they present so formidable an appearance at first sight, that they may be taken for solid tumors; especially is this so when their walls are compact and well organized, nearly if not altogether obliterating the sense of fluctuation when pressure is made upon them.

Cases have come under the observation of the writer where it required the most delicate touch to detect any fluctuation when pressure was made upon the apex of the tumor.

In some cases the diagnosis cannot be determined accurately until after one or more teeth are removed that are involved with the tumor. After such operation, a probe carried through the alveolus will usually reveal the true condition of the lesion. One or more dead teeth are found involved—one, however, being the rule in most cases which have come to the notice of the writer, while two, and sometimes three, are implicated with the the tumor. The dead tooth may be easily distinguished from the living ones by its opaque appearance. Such tooth may be carious, and it may not.

Primarily the dentritic cyst originates from what pathologists call a "cold abscess," that is, an abscess which has never opened; subsequently, having developed into a tumor. The interior of the cyst has a fibrous lining, and being compact in structure, is the seat of an inflammatory process. The cyst contains a puriform fluid; it may attain such magnitude as to invest several teeth and extend beyond the alveolar process. The tumor is usually oval in shape, with its apex on a line with the diseased tooth directly involved. The size of the tumor may be as large as a hulled waluut or as small as hazel-nut; crepitates under pressure, and feels like parchment. In cases of long standing, considerable resorption of the alveolar process takes place, and the teeth immediately connected will be loose; especially will this be the case if the alveolar borders are broken; these teeth should be removed. These tumors are found painless, as a rule. I have met with cases, however, where an acute inflammatory condition was present, with all the symptoms of acute periodontitis manifested. So that it could have been readily mistaken for the pointing of an alveolar abscess.

*Pathology.*—Cysts of the jaw may be either simple or compound; whether they be cysts of retention, exudation cysts, or extravasation cysts belonging to the jaws, is a matter not as yet fully established. The exudation cyst is a secretory cyst; in a generic relation, however, it is just the opposite of the retention cyst. Serous sacs form the foundations of the exudation cysts. "The mode of development of cysts of the jaws," says Wedl, "has not yet been determined; it therefore becomes necessary, in order to throw more light on the subject, to pursue further anatomical investigations in that direction."

Rindfleisch says: "The accumulation of the fluid is not produced by the continuance of the normal secretion, but by an exudation surpassing the normal measure of the serum of the blood with salts, albumen, fibrinogenous substance, and extractives, in the most varying proportions. The exudation cysts have little to do with pathological new formation. Of extravasation cysts," he says, "a parenchymatous bleeding can very well be the point of departure for the formation of a cyst. The hemorrhagic depot can present itself primarily as a cyst, namely, when the blood is poured out between two surfaces in themselves smooth; for example, bone and periosteum, cartilage and perichondrium, and thereafter remains fluid. As a cyst may also be formed when upon the one hand the limitary parenchyma furnishes a connective tissue membrane, upon the other hand, the blood itself is resorbed through a series of metamorphoses up to a small remainder, and is replaced by a clear fluid."

The above-mentioned condition is liable to manifest itself within the body of the jaw, the bone and periosteum, after severe mechanical injuries to the bone, and the rupture of blood-vessels within the parenchyma. There can be little doubt that many of the so-called dentritic cysts of the jaws have their origin primarily from causes brought about by falls, strokes and mechanical violence, causing rupture of blood-vessels. It is quite true, history of cases fully confirms such facts.

Clinical observations leads us to believe, however, that only in cases where the abscess does not open, we find the pathological new formation taking place within the jaws. Pulpitis, and as has been observed, followed by pericementitis and periodontis, is a prolific cause of the development of the dentritic cystic tumor.

*Treatment.*—The removal of all dead teeth involved. Other teeth whose pulps are living may be loose, and to a casual observer appear to be complicated, but a careful examination will reveal the fact that they should not be disturbed but retained in their places; only one tooth may be the offender, being a dead one which has caused the trouble. After the removal of the cause, let it be either one or more dead teeth or fangs of teeth, cyst walls may be punctured with a sharp instrument, and the contents of the sac released, this being done by carrying the instument through the alveoli, and not through the bony parietes of the jaw. After the contents of the sac is let out, and the sharp spicula of bone trimmed, with engine burs, tincture of iodine full strength may be forced into the cyst sac, by saturating tufts of cotton-wool and allowing them to remain, again repeating the treatment at intervals of a day. If necrosis of bone be present, it is good practice to alternate the iodine treatment with aromatic sulphuric acid. Cases generally yield to this treatment in from six week to three months. I have seen cases not yielding to treatment for nine months. There are other and shorter methods in the treatment which perhaps some would prefer—the cutting down through the body of the tumor, by making a crucial incision and scraping out the contents of the sac, afterwards allowing nature to do the rest—but I do not believe it is the best or safest way. There is surely a much greater loss of structure, which is never restored as in the former method by granulation, after the secreting cells have been destroyed by medicinal applications of iodine and sulphuric acid treatment.—*Medical and Surgical Reporter.*

